# Dark plumes of glacial meltwater affect vertical distribution of zooplankton in the Arctic

**DOI:** 10.1038/s41598-022-22475-8

**Published:** 2022-10-26

**Authors:** Marlena Szeligowska, Emilia Trudnowska, Rafał Boehnke, Katarzyna Błachowiak-Samołyk

**Affiliations:** Pelagic Biocenosis Functioning Laboratory, Marine Ecology Department, Institute of Oceanology, Polish Academy of Sciences, Sopot, Poland

**Keywords:** Biooceanography, Climate-change ecology, Ecosystem ecology, Boreal ecology, Marine biology

## Abstract

In polar regions, the release of glacial meltwater resulting in turbid plumes is expected to transform coastal waters with numerous consequences on the marine ecosystem. This study aimed to determine the influence of turbidity regimes on the vertical distribution of copepods together with their potential food (chlorophyll *a* fluorescence) and non-visual predators (gelatinous zooplankton). Hydrography, turbidity, suspended particulate matter and chlorophyll *a* were studied in July and August 2019 in West Spitsbergen waters (European Arctic). Fine-scale vertical distribution patterns of zooplankton were assessed by an optical counter (LOPC) and underwater camera (UVP) and verified by plankton nets. In waters with the shallow impact of dark plumes, *Calanus* spp. and gelatinous zooplankton were concentrated in the upper water layers, whereas in areas with a thick turbid layer, they were distributed evenly in the water column. However, chlorophyll *a* peaks were found to be restricted to the surface in the turbid waters and there were subsurface maxima in the shallow turbidity regime. Regardless of the region, the turbidity regime was a significant factor shaping the vertical distribution of *Calanus* spp. We speculate that similar trends might be observed in other rapidly emerging turbid ecosystems and urge that future plankton research should also include relatively simple turbidity measurements.

## Introduction

The consequences of global warming have an impact on marine ecosystems at both poles, leading to a cascade of changes in the physical environment, biogeochemical processes, and ecosystem functioning^1–3^. Sea ice loss results in increased light penetration in the water column during spring and a subsequent increase in ocean productivity^4^. However, the light available for primary production is reduced later in the year in coastal zones when glacial meltwater discharge is at its maximum^5^. During summer, light attenuation in the glacial bays is affected mainly by the process of light scattering by suspended particulate matter (SPM)^6^. Such water darkening (or shading) is caused by vast quantities of sediment transported from land into the coastal ocean, which produces turbid plumes in a form of ‘brown zones’. Importantly, glacial meltwater and sediment discharge have increased globally in recent decades, resulting in longer SPM residence time and higher accumulation rates^7,8^. Consequently, polar coastal ecosystems have already experienced regime shifts toward darker waters^9^ that are expected to advance in the future, particularly in the European Arctic, which is globally the most sensitive to an increased presence of warm Atlantic Water (AW)^10^ and both interannual and long-term climate dynamics^10^.

The understanding of the impacts that current changes have on near-glacial marine ecosystems can now be facilitated by new methods such as optical and imaging devices^11,12^, which are characterized by high resolutions that are comparable to the measurements of physical fields^13–15^. Such simultaneous studies are a key prerequisite for developing predictions about ecosystem response to environmental changes since intensified meltwater discharge creates variable small-scale environmental gradients. This may affect the structure of pelagic communities, plankton production rates^16^, and thus the amount and quality of food resources available for higher trophic levels, with consequences for both commercial fisheries and charismatic polar species^17–19^. In the Arctic, the sustainability of large stocks of ctenophores, fish, seabirds and marine mammals is dependent on copepods of the genus *Calanus*^20,21^, which is one of the best available Focal Ecosystem Components (FEC) for zooplankton monitoring^22^.

Most studies performed on glacier-influenced turbid waters have focused on horizontal gradients in zooplankton^11,12,17,23^, while their vertical distribution in Arctic fjords was mainly investigated in the context of diel and/or seasonal vertical migrations (DVM and SVM, respectively)^24–27^. During the Arctic summer, part of the zooplankton community performs a seasonal descent to deeper, more stable water layers to avoid starvation and predation pressure during winter, while the rest still feeds on phytoplankton in surface waters to accumulate sufficient lipid reserves for diapause^28,29^. Synchronized diel vertical migrations during the midnight sun were not observed in Arctic zooplankton^24–26^ and it is assumed that individuals respond to their own needs rather than as a population. Thus, zooplankton still feeding in the upper water layer can be affected by turbid plumes that control the depth of the euphotic zone, which directly influences primary production^30^ as well as phytoplankton composition and distribution^11^. Consequently, it is expected to force primarily herbivorous zooplankton such as *Calanus* spp. to regulate their vertical position to optimize feeding conditions. Zooplankton can also change the depth at which they reside due to a hard-wired response to light for predator avoidance^31^. However, in glacial bays, *Calanus* spp. might be less affected by visual predators, i.e. planktivorous fish and pursuit-diving seabirds, since high turbidity prevents prey detection, so they prefer to forage in clear waters away from the glacier fronts^19^. Tactile predators (i.e. gelatinous zooplankton) are also hindered due to e.g. clogging of their tentacles by marine aggregates^11^, and thus they might not be as successful relative to visual predators^32^ as it was previously shown in Norwegian fjords^33^. Additionally, the vertical positioning of zooplankton may be influenced by upwelling, as meltwater at the fronts of marine-terminating glaciers enters the marine environment at depth and forms a buoyant plume that transports zooplankton to the surface. However, they may reach the surface at a distance from the glacier front depending on the density of the plume^34^. On the other hand, estuarine circulation, which is observed in Svalbard fjords, may entrap both advected and local zooplankton populations at specific depths of the inner bays^35^.

Here, we present a study on the influence of various vertical ranges of turbid meltwater plumes on the vertical distribution of zooplankton in West Spitsbergen coastal waters (Svalbard archipelago, European Arctic) during the midnight sun (July and August 2019), when meltwater discharge is at its maximum and turbid glacial plumes create ‘brown zones’. To reduce potential bias caused by the diel light cycle, we selected measurements performed between 6:00 and 19:00 Local Time (LT)^36,37^. In four areas affected by glacier melting, we used an optical particle counter (Laser Optical Plankton Counter, LOPC) and an in-situ camera (Underwater Vision Profiler, UVP) to study the vertical distribution of marine particles as well as copepods and gelatinous zooplankton, which was verified by traditional sampling with nets (WP2). We also studied hydrography, turbidity, suspended particulate matter, fluorescence and concentration of chlorophyll *a*. Our aim was to test the hypothesis that the thickness of the turbid water layer shapes different vertical distribution patterns of primarily herbivorous *Calanus* spp. We also verified if the position of their food (chlorophyll proxy) and predators (gelatinous zooplankton) was respectively altered.

## Materials and methods

### Sampling design and study area

**Figure 1:**
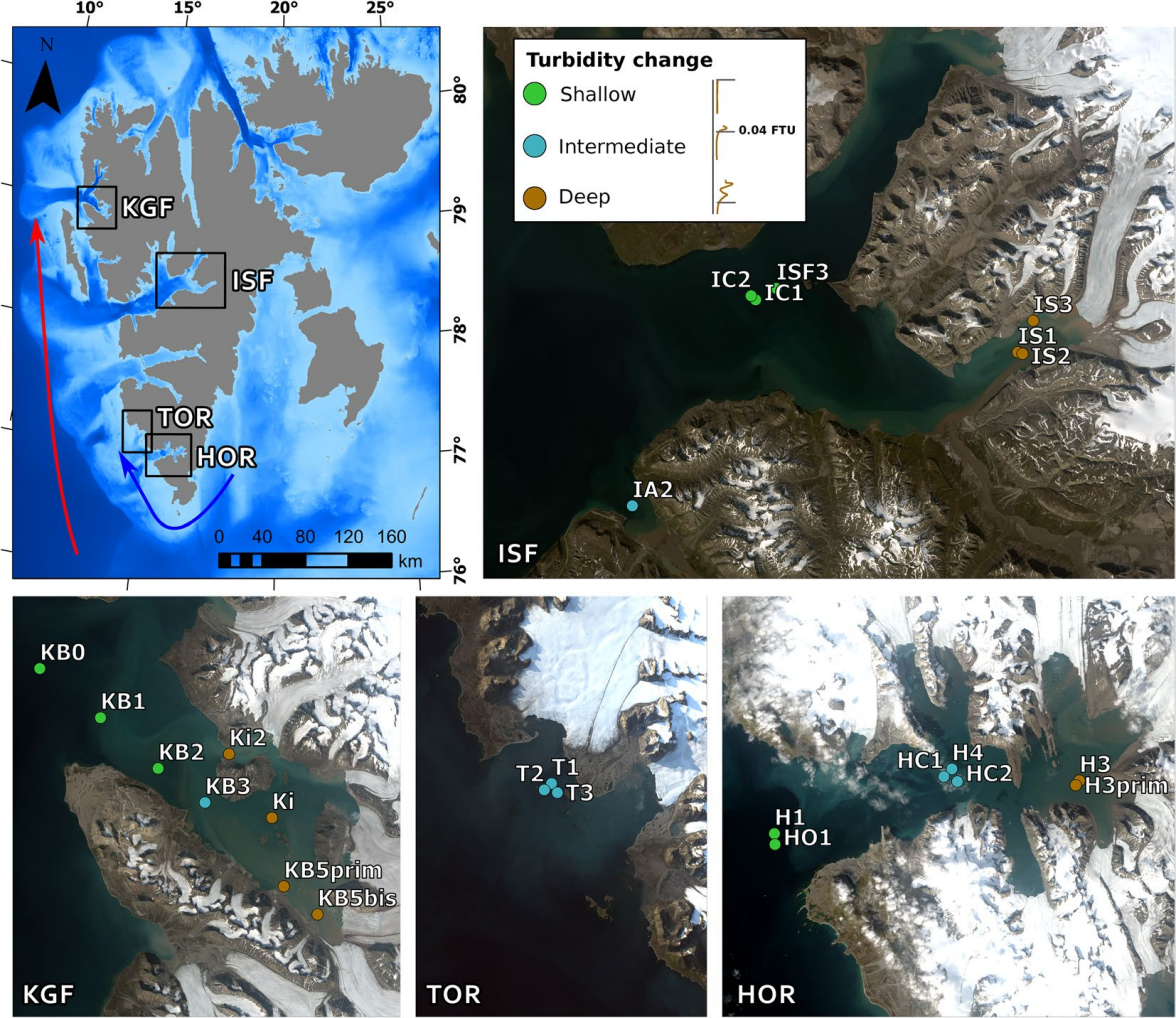
Map of the Svalbard Archipelago with studied West Spitsbergen regions (*ISF* Isfjorden, *KGF* Kongsfjorden, *TOR* Torellbreen, *HOR* Hornsund)— Landsat satellite images from summer 2019 (downloaded from https://glovis.usgs.gov/app and generated in ArcGIS Pro 2.8.0). The color-coding with the examples of turbidity profiles indicates the depth of turbidity change (shallow < 10 m—green, intermediate—blue, deep > 40 m—brown). The red arrow represents Atlantic Water and the blue arrow represents Arctic Water.

Data were collected in the West Spitsbergen coastal waters (Fig. 1), during a cruise on R/V Oceania conducted by the Institute of Oceanology Polish Academy of Sciences (IO PAN) in late July and early August of 2019 at 25 sampling stations in four regions (Table 1). Despite midnight sun conditions and the fact that we did not observe any signs of even weak upward movement characteristic for DVM during night hours, we narrowed our analysis into measurements performed during the daytime between 6:00 and 19:00 LT with exception of two stations (KB5prim and KB5bis), which were sampled around 22:00 LT. All stations were assigned a factor based on the transition from high to low turbidity (threshold 0.04 FTU, formazin turbidity units, Fig. 1), with a Shallow (S) regime for depth of turbidity change < 10 m, Intermediate (I) for depth 10–40 m and Deep (D) for depth > 40 m. This threshold was defined based on well-pronounced inflection points in turbidity profiles in the Intermediate regime. It was introduced as a proxy of a transition zone between the darkened surface water layer with a high concentration of fine sediment (so-called glacial flour) and the water layer below. This transition zone was observed in our previous study in Isfjorden in drop camera images (Fig. 4A in Szeligowska et al., 2021)^11^. The article included data that are also part of this study (7 stations in Isfjorden) such as distribution of environmental parameters (temperature, salinity, turbidity, chlorophyll *a* fluorescence and concentration, SPM and marine snow concentration, %PAR) and concentration of gelatinous zooplankton, mesozooplankton, and protists integrated for upper 50 m. This previous study allowed us to describe the main features of the interplay between plankton and particles transported by meltwater in Isfjorden in a horizontal manner. In the current article, we also consider other West Spitsbergen fjords and focus on *Calanus* spp. and copepods measurements by LOPC and UVP, respectively, to infer vertical patterns of zooplankton that are more complex in nature.

**Table 1:** Details of sampling and measurements carried out in the West Spitsbergen coastal waters in 2019 with turbidity regimes assigned according to the depth of turbidity change (m) with a 0.04 FTU threshold.

Station	Region	Regime	Turbidity change (m)	Mean turbidity	Max depth (m)	Date	Local Time (UTC + 2)	Latitude (N)	Longitude (E)
IS1	Isfjorden	D	71.6	0.16	74	7/27/2019	10:30	78°24.594	17°06.055
IS2	Isfjorden	D	77.7	0.15	80	7/27/2019	11:15	78°24.533	17°07.239
IS3	Isfjorden	D	69.7	0.16	93	7/27/2019	13:08	78°26.226	17°09.345
ISF3	Isfjorden	S	9.6	0.02	93	7/27/2019	15:45	78°26.863	16°04.943
IC1	Isfjorden	S	4.5	0.02	150	7/27/2019	17:20	78°26.226	16°00.041
IC2	Isfjorden	S	6.5	0.02	152	7/27/2019	18:20	78°26.400	15°58.854
IA2	Isfjorden	I	28.5	0.06	80	7/28/2019	7:50	78°15.449	15°34.248
T1	Torellbreen	I	14.5	0.05	42	7/29/2019	16:10	77°09.462	14°43.342
T2	Torellbreen	I	14.5	0.04	52	7/29/2019	17:15	77°09.136	14°41.816
T3	Torellbreen	I	13.5	0.04	52	7/29/2019	18:10	77°09.064	14°44.811
H1	Hornsund	S	0	0.02	168	8/1/2019	7:57	76°56.057	15°21.947
HO1	Hornsund	S	0	0.02	150	8/1/2019	15:40	76°55.481	15°22.325
H4	Hornsund	I	28.5	0.09	106	8/3/2019	9:10	77°00.072	15°59.780
HC1	Hornsund	I	14.4	0.06	122	8/3/2019	16:20	76°59.663	15°58.162
HC2	Hornsund	I	30.5	0.06	105	8/3/2019	17:50	76°59.430	16°01.205
H3	Hornsund	D	67.5	0.11	120	8/4/2019	8:45	77°00.017	16°28.152
H3prim	Hornsund	D	60.4	0.09	118	8/4/2019	10:45	76°59.731	16°27.446
KB3	Kongsfjorden	I	10.6	0.06	341	8/7/2019	14:00	78°57.293	11°56.536
KB0	Kongsfjorden	S	3.5	0.02	322	8/9/2019	9:55	79°02.581	11°08.191
KB5prim	Kongsfjorden	D	49.5	0.43	50	8/9/2019	21:50*	78°53.775	12°20.041
KI	Kongsfjorden	D	76.3	0.13	79	8/10/2019	8:35	78°53.775	12°20.041
KI2	Kongsfjorden	D	59.5	0.11	60	8/10/2019	15:00	78°59.894	12°00.753
KB1	Kongsfjorden	S	6.5	0.03	335	8/11/2019	15:30	79°00.679	11°26.015
KB2	Kongsfjorden	S	8.5	0.02	307	8/11/2019	19:00	78°58.612	11°43.099
KB5bis	Kongsfjorden	D	60	0.26	64	8/11/2019	22:15*	78°52.635	12°29.789

A shallow (S) regime was assigned for depth of turbidity change < 10 m, intermediate (I) for 10–40 m, and deep (D) for depth > 40 m. Sampling time was restricted from 4:00 to 17:00 UTC (6:00–19:00 local time), except for two points where measurements were conducted around 22:00 LT (*).

West Spitsbergen fjords are glacial systems that are used as monitoring sites of climate-related changes and their possible impacts on biodiversity in the Arctic region^19,38^. They are increasingly influenced by freshwater, both from glacial and riverine discharge. Moreover, Isfjorden (ISF) and Kongsfjorden (KGF) have wide openings and are highly exposed to the influx of warm and saline AW (Fig. 1, red arrow)^39,40^. Hornsund (HOR) and Torellbreen (TOR), which are the southernmost regions, are strongly influenced by the colder and less saline coastal Sørkapp Current that transports Arctic Water (Fig. 1, blue arrow)^41^.

### Sampling and measurements

Vertical measurements of the Laser Optical Plankton Counter-Conductivity-Temperature-Depth-Fluorescence-Turbidity (LOPC-CTD-F-T) platform provided data on environmental parameters (temperature, salinity, turbidity, chlorophyll *a* fluorescence, and particle concentrations), and preceded the deployment of the UVP and collection of zooplankton by nets.

Salinity and temperature were measured with conductivity-temperature-depth sensors (CTD, SBE 911plus, Seabird Electronics Inc., USA), water turbidity was assessed by a turbidity meter (Seapoint Sensors Inc., USA) and quantified in formazin turbidity units (FTU), whereas fluorescence of chlorophyll *a* was measured with a fluorometer (Seapoint Sensors Inc., USA). The concentration of particles and plankton was measured by a laser optical plankton counter LOPC (Brooke Ocean Technology Dartmouth, Canada). The working principles of LOPC have been described in other studies^42–44^. The measurements of the LOPC-CTD-F-T platform were averaged over 1 m depth intervals. Measurements from the upper 1 m depth were discarded to minimize errors due to wave action, stray light or air bubble formation. The concentration of particles was calculated as the counts per cubic meter (individuals·m^−3^) based on the volume of the sampled water. The category of *Calanus* was selected based on size (1–2.5 mm equivalent spherical diameter, ESD)^44,45^ and opacity (attenuance index > 0.4)^46^ parameters that were calibrated for the older life stages of *Calanus*.

Light distributions in the water column were calculated from vertical profiles of spectral downward irradiance, Ed (z, λ), made with a high-performance free-fall aquatic profiler C-OPS (Compact Optical Profiling System, Biospherical Instruments, Inc.). A C-OPS radiometer equipped with 19 wavebands (from 305 to 765 nm, with a separate dedicated PAR channel) was mounted in a free-fall backplane, allowing deployment far from the ship’s shadow. The instrument was equipped with a reference irradiance sensor, mounted on the deck to measure incident irradiance and control the stability of ambient light during deployment. From those measurements, the depth of the euphotic zone (1% surface PAR) was determined.

Seawater samples were collected by 8 L Niskin bottles from five depths (surface, 5 m, 15 m, 25 m and 40 m). The total, inorganic and organic mass of SPM were determined gravimetrically^47^ from seawater subsamples collected at 13 stations (IS2, IC1, T1, H1, H4, H3, KB0, KI, KI2, KB1, KB2, KB5prim, KB5bis). Three replicates were carried out at each station. The coefficient of variation, which defined the reproducibility of the replicates, was 9.5% on average.

To estimate chlorophyll *a* concentrations, 250–400 mL of the subsamples were filtered through GF/F Whatman filters (pore size 0.7 μm) and immediately frozen at − 80 °C. The samples were extracted for 24 h in 8 mL of 96% ethanol in darkness at room temperature. Chlorophyll *a* concentration was determined by a Perkin Elmer Lambda 650 spectrophotometer. The optical density (∆OD) of the extract at 665 nm was corrected for background absorbance in the near-infrared radiation (750 nm) and converted to chlorophyll *a* concentration using the following equation:

$${chl}_{a}=\frac{{10}^{3}\cdot \Delta OD\cdot {V}_{E}}{83\cdot {V}_{W}\cdot l}$$

which included volume of filtered seawater (V_w_) and ethanol extract (V_E_), a 2-cm path length of the cuvette (l), and the chlorophyll *a*-specific absorption coefficient in 96% ethanol^48,49^.

The abundance of copepods and gelatinous zooplankton was measured using the high-definition and high-frequency UVP camera (5hd; Hydroptic, France)^50^. The UVP system detects and counts all objects larger than ~ 100 μm in a defined and illuminated volume of ~ 1 dm^3^ (cubic decimeter), and automatically stores cut-out vignettes of objects > 30 pixels (approximately 500 μm). Due to technical problems, UVP was not utilized at some stations (IS1, IS2, IS3, T1, KB2, and KB5bis). Images recorded by UVP were validated manually in the Ecotaxa web application (https://ecotaxa.obs-vlfr.fr/).

Zooplankton sampling was conducted in three stratified vertical hauls (bottom–50, 50–10 and 10–0 m) of WP2 net with a mesh size of 100 μm and an opening area of 0.25 m^2^. Such depth layers were set in order to represent (i) the layer most affected by turbid plumes (< 10 m), (ii) the layer of the highest production, and thus the highest concentrations of zooplankton during summer (< 50 m) and (iii) the deep water refugium (> 50). The assumed filtration efficiency of the net was 100%. Samples were immediately preserved with a 4% borax-buffered formaldehyde-seawater solution. Prior to quantitative and qualitative analyses, macrozooplankton were removed and identified separately. Later, zooplankton samples were prepared in appropriate volumes depending on their densities; subsequently, 2 mL subsamples were taken using a Henson-Stempel pipette. The analysis was conducted under a Leica M80 stereo microscope at 10–40× magnifications. A minimum of 300 organisms were enumerated and identified from subsamples. The remainder of each sample was analyzed to list and enumerate life stages and species that did not get caught in the subsamples. Zooplankton abundance was calculated as the number of individuals per cubic meter (individuals·m^−3^). Zooplankton was not collected at KB2 and KB3 stations.

We did not separate *Calanus* spp. into species based on size, since this classification should be interpreted with caution. *Calanus* spp. individuals are much smaller in glacial bays, leading to the most cases of misidentification, which was recently well-documented in both Isfjorden and Hornsund^51,52^. However, genetic identification from West Spitsbergen fjords showed similar population age structure of the two co-occurring species (*C. glacialis* and *C. finmarchicus*)^52^.

### Data analysis and statistics

Landsat8 composite RGB images of the investigated regions were prepared using 4-3-2 spectral bands (spatial resolution of 30 m) of LC08_L1TP_216003_20190727_20190801_01_T1, LC08_L1TP_216004_20190727_20190801_01_T1 and LC08_L1TP_031239_20190812_20190820_01_T1 downloaded from https://glovis.usgs.gov/app. Bathymetric data were taken from IBCAO V4.1 Grid (200 m × 200 m grid cell spacing)^53^. The map and images were generated in ArcGIS Pro 2.8.0.

The plots were generated in Python 3.7^54^ using Matplotlib 3.1.1^55^^,^^56^, seaborn 0.11.1, and Pandas 1.0.5^57^^,^^58^, and arranged in Inkscape 0.92.4.

Brunt-Väisälä frequency squared (N^2^), which is a measure of the vertical stratification or the static stability of the water column, was calculated from salinity, temperature, and pressure using the MATLAB seawater toolbox (http://mooring.ucsd.edu/software/matlab/doc/toolbox/ocean/swstate.html). If N^2^ > 0, the water column is hydrostatically stable, and when N^2^ < 0, it becomes hydrostatically unstable^59^. The level of stratification can be based on the following criteria: N^2^ < 2·10^−5^ rad^2^ s^−2^ corresponds to non-stratified, 2·10^−5^ rad^2^s^−2^ < N^2^ < 5·10^−5^ rad^2^ s^−2^ corresponds to weakly stratified and N^2^ > 5·10^−5^ rad^2^ s^−2^ corresponds to strongly stratified^60^.

We calculated the mean abundance of *Calanus* (LOPC) and copepods (UVP) within three water layers (bottom–50, 50–10 and 10–0 m) to compare it with zooplankton sampling by WP2 net. To assess the positioning of *Calanus* spp. and copepods within the 0–50 m water layer, we calculated the weighted mean depth with the abundance *of Calanus* spp. (LOPC) and copepods (UVP) treated as weights.

To test if selected variables differed significantly between defined regimes (Shallow, Intermediate, Deep), we used the non-parametric Kruskal–Wallis and post-hoc Dunn tests. To assess the similarities in environmental parameters or *Calanus* spp. life stage compositions among the stations and water layers, a non-metric multidimensional scaling (nMDS) analysis was used. The environmental parameters (salinity, temperature, turbidity, and fluorescence of chlorophyll *a*) averaged for the surface (< 10 m), intermediate (10–50 m) and deep (> 50 m) water layers, as well as station-specific variables (maximum turbidity, maximum fluorescence, and depths for these maxima) (Supp. Table 1) were analyzed on previously normalized Euclidian distance resemblance data. The *Calanus* spp. life stage and nauplii abundances in the three water layers (Supp. Table 2) were log-transformed to ensure a balanced view of the community structure. The analysis of *Calanus* spp. life stage composition was based on the Bray–Curtis similarity matrices to reflect the resemblance among communities at different stations and layers. To test the hypothesis of no differences in *Calanus* spp. community structure among these three groups (S, I, D) and regions (KGF, ISF, TOR, HOR), we used PERMANOVA and pair-wise comparisons with 9999 permutations. The pair-wise test was repeated three times as a precaution not to reject a true null hypothesis.

We applied a redundancy analysis (RDA) to study the relationships between observed variation in biological and environmental data, namely *Calanus* spp. developmental stage composition and *Calanus* weighted mean depth were tested in relation to environmental and spatial factors. Variation partitioning to the RDA results was applied to divide the influence of environmental factors and the effects of the spatial location of sampling stations on both *Calanus* datasets. For *Calanus* spp. life stage composition variability, we used 1 set of response variables (*Calanus* spp. life stage composition) and 2 sets of predictors: environmental variables (sampled layer thickness, salinity, temperature, turbidity, and fluorescence of chlorophyll *a* averaged for three water layers, maximum turbidity, maximum fluorescence and depths for these maxima) and spatial characteristics of sampling stations (latitude, longitude, and bottom depth). For *Calanus* weighted mean depth in the upper 50 m, we used 1 set of response variables (*Calanus* weighted mean depth in the upper 50 m) and 3 sets of predictors: environmental variables related to turbidity (mean turbidity, maximum turbidity and depth for this maximum), other environmental parameters (salinity, temperature, fluorescence of chlorophyll *a*, maximum fluorescence and depth for this maximum) and spatial characteristics of sampling stations (latitude, longitude, and bottom depth). The statistical analyses were computed in PRIMER v7 and PERMANOVA^61,62^, scipy 1.5.2^63^ and scikit-posthocs 0.6.7^64^.

## Results

### Environmental conditions

**Figure 2:**
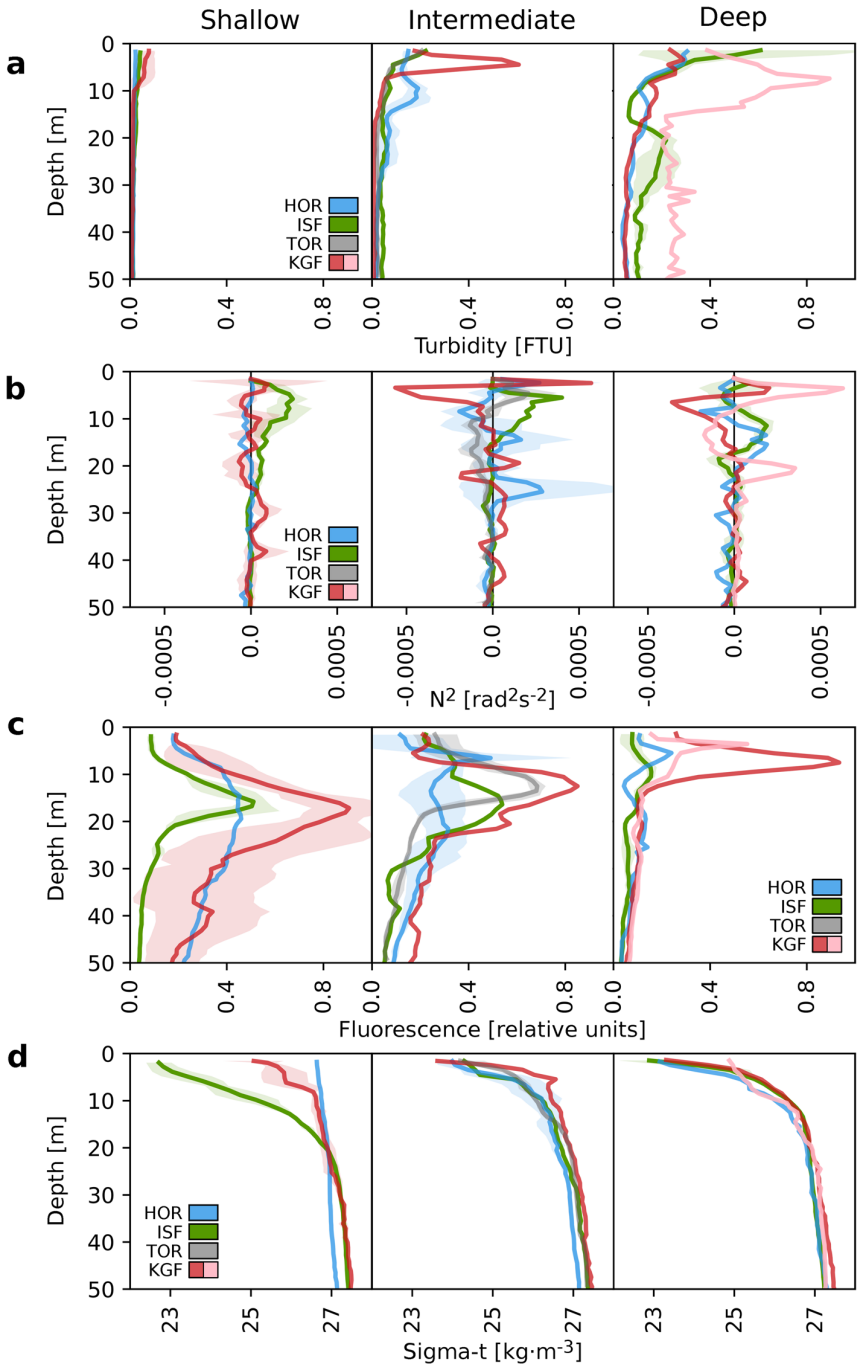
Vertical profiles of turbidity (FTU) (**a**), water column stability N^2^ (rad^2^ s^−2^) (**b**), fluorescence of chlorophyll *a* (relative units) (**c**) and water density (sigma-t, kg·m^−3^) (**d**) averaged over areas (lines) with standard deviation (shadows) in three regimes of the depth of turbidity change. The color-coding indicates regions: Hornsund (HOR, blue), Isfjorden (ISF, green), Torellbreen (TOR, grey), and Kongsfjorden (KGF, red; in the Deep regime: KI and KI2, red; KB5prim and KB5bis, pink).

Three different regimes were established based on a vertical range of turbidity referred to as depth of turbidity change. In the Shallow regime, turbidity was higher than 0.04 FTU only at the surface (within 10 m water layer) (Fig. 2a), water masses were stable (N^2^ > 0) (Fig. 2b) and wide subsurface chlorophyll *a* maxima were observed (Fig. 2c). In the Intermediate regime, turbidity was still higher than 0.04 FTU within 10–40 m water layer (Fig. 2a) and the fluorescence of chlorophyll *a* was higher than 0.2 relative units only down to around 25 m (Fig. 2c). In the Deep regime, turbidity > 0.04 FTU had the highest vertical range, extending over most of the water column (> 40 m), whereas fluorescence peaks were restricted to only the upper 10 m water layer. Interestingly, subsurface turbidity peaks in the 10–20 m water layer coincided with a bimodal distribution of fluorescence of chlorophyll *a* (e.g. Intermediate and Deep regime in HOR). The highest turbidity was measured close to the glacier fronts in KGF, whereas the lowest fluorescence of chlorophyll *a* was in HOR and ISF in the Deep regime. Plumes of glacial meltwater in Intermediate and Deep regimes destabilized the water column. They decreased the temperature (around 2 °C decrease) and salinity (around 5 units) (Supp. Fig. 1). Water density (sigma-t) was lower at the surface than at 20 m depth (increasing from around 23 to 27 kg·m^−3^) in all regions except for Shallow regime in Hornsund (Fig. 2d).

**Figure 3:**
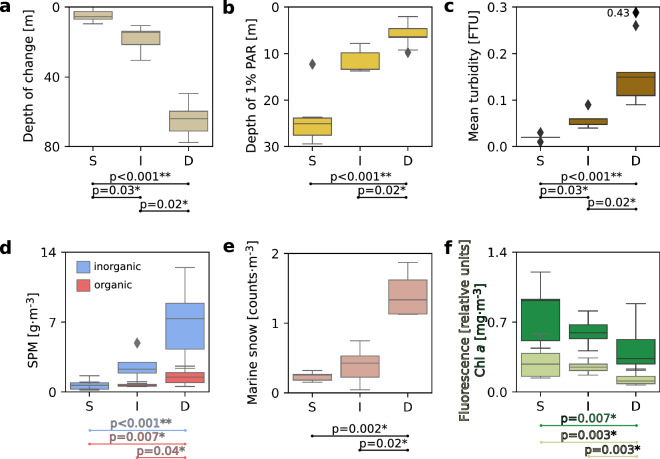
Boxplots presenting differences in median values (with quartiles) of environmental conditions in three regimes of the depth of turbidity change (S—shallow, I—intermediate, D—deep): depth of turbidity change (m, beige) (**a**), depth of euphotic zone (1% PAR, m, yellow) (**b**), mean turbidity in 50 m water layer (FTU, brown) (**c**), SPM concentration at the surface (0–5 m, g·m^−3^, inorganic—blue, organic—red) (**d**), mean marine snow concentration at stations (coutns·m^−3^, pink) (**e**), mean fluorescence in 50 m water layer (relative units, light green) and integrated chlorophyll *a* concentration (mg·m^−3^, dark green) (**f**) with p values for significant differences (post-hoc Dunn test, * indicates p < 0.05, ** indicates p < 0.001).

The thickness of the turbid water layer reached up to 80 m in the Deep regime (Fig. 3a), and the depth of the euphotic zone (1% PAR) decreased significantly from around 25 m in the Shallow regime to around 7 m in the Deep regime (H = 17, p < 0.001 for Kruskal–Wallis test, see Fig. 3b for post-hoc Dunn test results). The depth of turbidity change (Fig. 3a), mean turbidity in the upper 50 m water layer (Fig. 3c), concentration of inorganic SPM at the surface (Fig. 3d), and concentration of marine snow (Fig. 3e) were the highest in Deep regime and significantly different among regimes (H = 21, p < 0.001; H = 21, p < 0.001; H = 17, p < 0.001; H = 10, p = 0.008 for Kruskal–Wallis test, respectively, see Fig. 3 for post-hoc Dunn test results). The concentration of organic SPM was only slightly higher at stations with Deep turbidity change in comparison to the Shallow and Intermediate regimes (H = 8.74, p = 0.01 for Kruskal–Wallis test). Mean fluorescence in the upper 50 m water layer and concentration of chlorophyll *a* were high in Intermediate and Shallow (up to 1.2 mg·m^−3^) and significantly lower in the Deep regime (H = 13, p = 0.002, and H = 7, p = 0.02 for Kruskal–Wallis test, respectively, see Fig. 3f for post-hoc Dunn test results).

### Fine-scale measurements of copepods, *Calanus* spp. and gelatinous zooplankton

**Figure 4:**
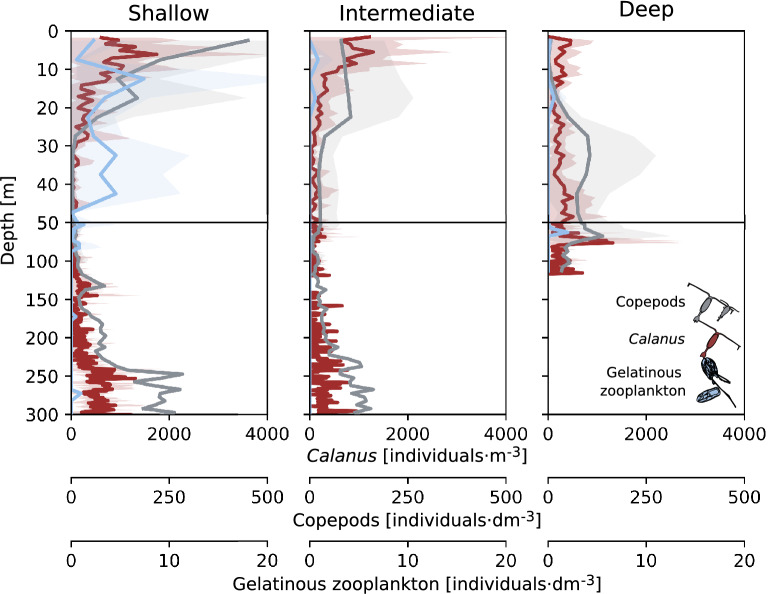
Vertical profiles of the abundance of copepods (individuals·dm^−3^, UVP, grey), *Calanus* spp. (individuals·m^−3^, LOPC, red) and gelatinous zooplankton (individuals·dm^−3^, UVP, blue), averaged over regimes (lines) with standard deviation (shadows) in three regimes of the depth of turbidity change.

**Figure 5:**
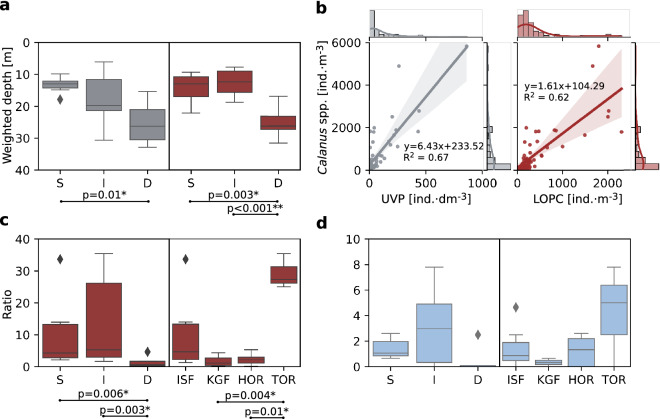
(**a**) Weighted mean depth for copepods (UVP, grey) and *Calanus* (LOPC, red) in the 50 m water layer. (**b**) Correlation between UVP and LOPC measurements with *Calanus* spp. (plankton net) with marginal histograms and density plots. Vertical positioning of *Calanus* spp. (**c**) and gelatinous zooplankton (**d**) displayed as a ratio of abundance (individuals·m^−3^, plankton net) in the 0–10 and 10–50 m water layers in three regimes of the depth of turbidity change (S—shallow, I—intermediate, D—deep) and regions (*ISF* Isfjorden, *KGF* Kongsfjorden, *HOR* Hornsund, *TOR* Torellbreen).

The highest abundance of copepods (up to 1000 ind.·dm^−3^, UVP), *Calanus* spp. (up to 5000 ind.·m^−3^, LOPC) and gelatinous zooplankton (mostly *Mertensia ovum*, up to 20 ind.·dm^−3^, UVP) was measured in the surface water layer (down to around 25 m) in Shallow and Intermediate regimes (Fig. 4 and Supp. Fig. 2). In the Deep regime, these surface peaks were not observed, and *Calanus* spp. and copepods were relatively evenly distributed in the whole water column, whereas gelatinous zooplankton were often not observed. The weighted mean depth of copepods (UVP) and *Calanus* spp. (LOPC) within the 50 m water layer showed that they resided significantly deeper in the water column in the Deep regime (H = 6, p = 0.04, and H = 15, p < 0.001 for Kruskal–Wallis test, respectively, see Fig. 5a for post-hoc Dunn test results). In all regimes, there was an increase in *Calanus* spp. and copepods abundance near the sea bottom (Fig. 4). In this deepest water layer (50 m–bottom), *Calanus* spp. population consisted of around 90% of CV and CIV copepodite stages, while in the upper 50 m it was usually around 70–80% due to a relatively high abundance of younger stages (Supp. Fig. 3). Also, in Shallow and Intermediate regimes, CI–CIII stages were relatively abundant within the 0–50 water layer (constituted 15–30% of the population), whereas in the Deep regime they were concentrated within 0–10 m (35%). The vertical distribution patterns of *Calanus* spp. assessed via LOPC well-matched the distribution patterns of copepods recorded by UVP (Fig. 4). Also, the correlation between UVP measurements and *Calanus* spp. collected by plankton net as well as between LOPC measurements and *Calanus* spp. collected by plankton net was high (R^2^ = 0.67 and 0.62, respectively, Fig. 5b). However, there was no correlation between the general category ‘copepods’ measured by UVP and total copepods abundance collected by plankton net (see Supp. Figs. 2 and 6a). For the ratio of total *Calanus* spp. abundance (plankton net) in the 0–10 m to 10–50 m water layers, there were statistically significant differences between Shallow and Deep as well as the Intermediate and Deep regimes (H = 10.94, p = 0.004 for Kruskal–Wallis test, see Fig. 5c for post-hoc Dunn test results), which is in line with the weighted mean depth of *Calanus* spp. (UVP). The ratio was also significantly higher in TOR than in HOR and KGF. Patterns of ratios of gelatinous zooplankton between water layers followed patterns of *Calanus* spp., though there were no significant differences between regimes and regions (Fig. 5d).

### Multidimensional scaling—regimes of turbidity

The community structure of *Calanus* spp. in the 0–10 m and 10–50 m water layers differed statistically among darkening regimes and regions, but the interaction of those two factors was not significant for the upper layer (Permanova, see Supp. Fig. 4 for statistics). The Shallow and Deep regimes as well as the Deep and Intermediate regimes differed from each other in these two layers, whereas there were no differences for pair-wise comparisons between the Shallow and Intermediate regimes. In the 50–bottom water layer, only the region was shaping community structure of *Calanus* spp. The multidimensional scaling of environmental parameters and *Calanus* spp. age structure grouped data points according to their affiliations to the regime of turbidity change (Fig. 7a,b). The mean turbidity in the water layers (0–10, 10–50, 50–bottom) was increasing from stations with a Shallow depth of turbidity change to the ones with a Deep depth of turbidity change. Within water layers shaped by the regime (0–10, 10–50 m), the abundance of *Calanus* spp. CV decreased with salinity, maximum turbidity and depth of maximum turbidity, and increased with temperature, mean fluorescence, maximum fluorescence and depth of maximum fluorescence. Also, in the Shallow and Intermediate regimes abundance of *Calanus* spp., CV was higher in the 0–10 m water layer than in 10–50 m, while it was comparable or lower in the Deep regime. The RDA model with two groups of predictors explained 56.3% of the total variation in *Calanus* spp. developmental stage composition. The variance partitioning indicated that the effect of environmental variables was much stronger (36.3%) than the characteristics of the location of sampling stations (4.4%) and interactions between these groups of predictors (15.7%) (Fig. 7c). Moreover, the RDA model was even stronger in explaining *Calanus* spp. weighted mean depth (73.8% of the total variation) (Fig. 7d). Here, the variance partitioning indicated a dominating effect of environmental variables associated with turbidity (19.1%,) compared to other environmental variables (10.3%) and characteristics of location the of sampling (4.3%). However, the joint effect of these three sets of variables was the most important in explaining the variation (27.6%).

## Discussion

Processes responsible for zooplankton niche selection are important factors controlling the structure and dynamics of marine food webs, which shape the overall functioning of pelagic ecosystems. A better understanding and parametrization of these processes is necessary for the development of predictive models of biogeochemical cycles in the ocean. Still, little is known about the potential implications of global climate change on zooplankton vertical distribution, which in turn shapes the export and subsequent strength of the biological carbon pump^65,66^. Previous investigations have shown that during the Arctic summer, the majority of zooplankton biomass concentrates close to the surface^24,67,68^. Also, they reported asynchronous migration during the midnight sun, where each individual migrates according to its own needs rather than as the entire population performing diel vertical migrations^25^. More detailed zooplankton vertical positioning under various environmental settings has scarcely been studied, mostly due to methodological limitations. However, the development of high-resolution measurement tools, such as the optical counters and underwater camera systems used in this study, enabled us to follow small–scale distribution patterns of *Calanus* spp. Here we present an alteration of the typical vertical distribution of *Calanus* spp. in areas strongly affected by glacial plumes (Deep regime) in comparison to less affected areas (Shallow and Intermediate regime). Regardless of the differences in *Calanus* spp. community between four regions located along the way of the West Spitsbergen Current, the depth of turbidity change was a significant factor shaping their vertical distribution (Supp. Fig. 4, Fig. 7). These methods also allowed us to take into account the effects that the discharge of dark meltwater plumes might have on tactile predators.

### Environmental conditions

Stations with a Shallow turbidity regime were selected as reference sites, where oceanographic conditions were favorable for typical summer *Calanus* spp. distribution, i.e. they were not directly affected by dark meltwater plumes and were characterized by the highest chlorophyll *a* concentrations, which can be treated as a food base proxy for *Calanus* spp. Wide subsurface chlorophyll *a* maxima developed in regions where the water column was unstable (HOR), whereas a stratified water column imposed counteracting effects on phytoplankton by favoring their suspension within the photic zone (ISF, KGF)^69^ (Fig. 2). In the Intermediate and Deep regimes, freshwater glacial plumes reduced both temperature and salinity, and affected substantial parts of the water column in the glacial bays. Due to this discharge, the concentration of inorganic SPM was higher in the Intermediate and Deep regimes than in the Shallow regime (Fig. 3d), whereas the depth of the euphotic zone was reduced (Fig. 3b). This is consistent with previous studies from Arctic coastal waters reporting that glacial meltwater mainly brings fine mineral particles (so-called glacial flour) that lead to increases in turbidity and changes in irradiance level^6,30,70,71^. As a consequence of altered underwater light conditions, the concentration of chlorophyll *a* was lowered, whereas peaks of fluorescence were shifted towards the surface, especially in the Deep regime with the lowest chlorophyll *a* concentrations confined to the upper 10 m (Fig. 2c). There were also areas with a bimodal distribution of fluorescence of chlorophyll *a* that could be caused by subsurface peaks of turbidity (10–20 m). Alternatively, subsurface plumes destabilize the water column and allow mixing, causing phytoplankton to sink^72^. Moreover, glacial discharge can support primary production in the Intermediate regime at depth depending on the buoyancy of the glacial plume, since meltwater outflow can also transport essential nutrients that might not be used in the glacial bays due to stratification constraints or light limitation.

### Typical vs. altered vertical patterns of *Calanus* spp. distribution in summer

**Figure 6:**
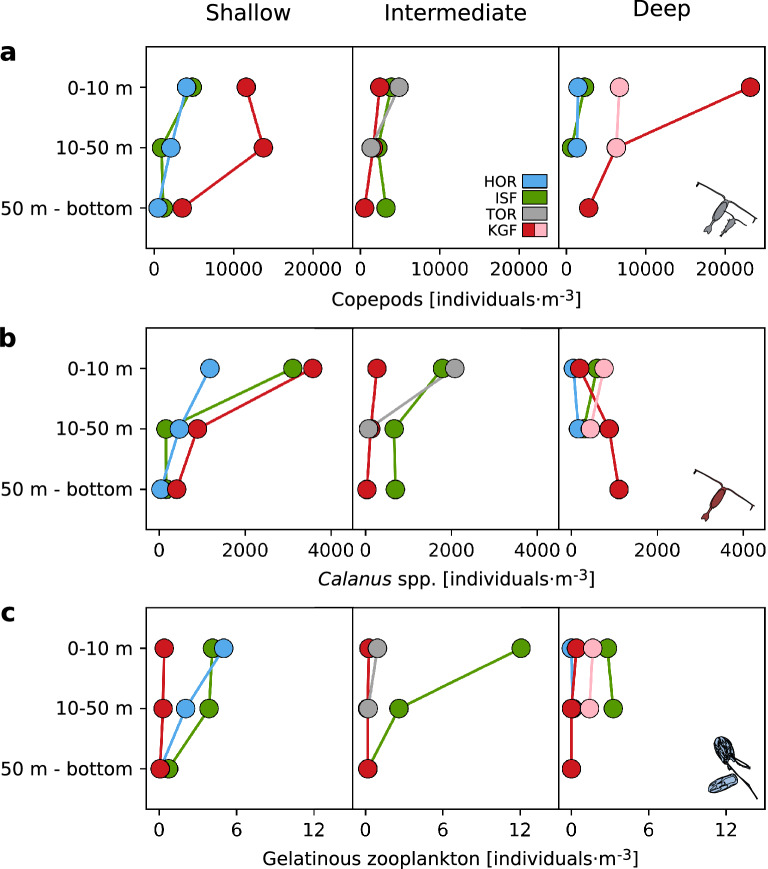
Abundance of copepods, *Calanus* spp., and gelatinous zooplankton (individuals·m^−3^, plankton net) averaged over areas in three regimes of the depth of turbidity change. The color-coding indicates regions: Hornsund (HOR, blue), Isfjorden (ISF, green), Torellbreen (TOR, grey), and Kongsfjorden (KGF, red; in the Deep regime: KI and KI2, red; KB5prim and KB5bis, pink).

In the Shallow regime, numerous *Calanus* spp./copepod individuals were observed to concentrate in the surface water layer (Figs. 4, 6). In the Intermediate regime, *Calanus* spp. were confined even closer to the surface, most likely due to the shallower depth of the euphotic zone, shallower depth of the chlorophyll *a* maxima, likely supply of essential nutrients (that could also increase the nutritional value of phytoplankton), and relaxation of visual predation. In the Deep regime, where a thick layer of high turbidity resulted in the lowest chlorophyll *a* concentrations, *Calanus* spp. was distributed evenly in the water column without the typical surface peak. It could be a result of (1) scarcity of food that is additionally dispersed by glacial meltwater, (2) increased risk of clogging of the filtering apparatus with fine suspended particles present at high concentration at the surface, (3) preference for feeding on marine aggregates that are formed below the most turbid layer from organic and inorganic particles and (4) avoidance of the fresh surface layer, since such low salinity may cause an osmotic shock. Moreover, *Calanus* spp. stunned by freshwater might be upwelled to the surface at the front of marine-terminating glaciers, and this mechanism could form local foraging grounds for surface feeders such as seabirds^34^ (not observed in this study), likely leading to a lower *Calanus* spp. abundance farther from the glacier (Deep regime). Additionally, the lack of a surface peak of *Calanus* spp. in the most turbid locations outside of an upwelling zone might in part be due to the outward transport of glacial meltwater. However, in open systems with deep sills such as Kongsfjorden, there is a strong entrapment mechanism due to the estuarine circulation induced by meltwater discharge as well as intensive advection of zooplankton with AW that can further shape the vertical structure of zooplankton. Indeed, we observed a high abundance of copepods at the surface even in the Deep regime in Kongsfjorden which was related to the high abundance of *Oithona similis* being one of the signs of strong AW transport. On the contrary, Hornsund and the inner branches of Isfjorden are more protected from AW inflow, and thus glacial processes seem to be dominant in shaping the vertical distribution of zooplankton there. However, according to our study, the vertical distribution of *Calanus* spp. was primarily shaped by turbidity regimes in a similar way in all the studied fjords.

There was a relatively high share of younger life stages (CI–CIII) in the upper 50 m water layer in the Shallow and Intermediate regimes, but only in the upper 10 m water layer in the Deep one (Supp. Fig. 3). This is probably due to the fact that they did not accumulate sufficient lipid reserves to sustain diapause and still had to continue feeding to reach older life stages^73,74^. Although *Calanus* spp. is regarded as primarily herbivorous, there are strong indications that it has flexible feeding strategies. When phytoplankton abundance is low, they can switch to alternative sources such as marine snow^52,75^, which was abundant in glacial bays (Fig. 3e), especially below the layer of highest turbidity. According to a previous study, both *C. glacialis* and *C. finmarchicus* had a mostly herbivorous, diatom-based diet in open oceanic waters, whereas in the glacial bay, they were shown to either feed on mineral particles transported from land or to starve^53^. The study reports that they were also more omnivorous close to glacial fronts, most likely due to decreased availability of phytoplankton and a typical summer shift from the domination of diatoms to cryptophytes that are less effectively grazed by zooplankton^52^. Thus, we suggest that the Intermediate regime represents suboptimal conditions where *Calanus* spp. can adapt to turbid plumes by moving closer to the surface to feed on phytoplankton without being easily spotted by visual predators. However, our results indicate that in the Deep regime, *Calanus* spp. alters their typical summer distribution patterns and becomes dispersed throughout the water column. It opens an interesting question of how such a mechanism will influence the ecology and evolution of this species in progressively darkening Arctic coastal waters^9^.

We speculate that *Calanus* spp. were concentrated close to the bottom in all investigated regions and regimes (Fig. 4), most likely because they began seasonal migration to start diapause (e.g. the population was represented mostly by the older life stages as shown on Suppl. Fig. 3). Such a descent to the deeper layers of the water column (usually > 100 m) is a strategy typically performed by high Arctic zooplankton^28,29^. This deep peak was previously observed when part of the summer−autumn *Calanus* spp. population was feeding in surface waters, while the rest resided in deep waters^76,77^. Already diapausing *Calanus* spp. most likely utilized the rich food base dominated by diatoms during the spring bloom when turbid plumes are not well developed. Moreover, in this season, primary production can be increased by meltwater nutrient input in glacial bays^78^ prior to light limitation due to SPM accumulation in summer.

### Vertical patterns of gelatinous zooplankton distribution

In the Shallow regime, the highest abundance of non-visual predators (mostly *M. ovum*) was observed, particularly in ISF. One possible scenario is that *Calanus* spp. abundance could have been reduced there (Figs. 4, 5c,d) considering the fact that ctenophores are extremely effective predators^79,80^, which could substantially impact the population of their prey. Strong associations between the observed vertical distributions of secondary consumers (gelatinous zooplankton) and *Calanus* spp. indicated that these carnivores followed their prey through the water column^28^ (Figs. 4, 5cd, 6).

**Figure 7:**
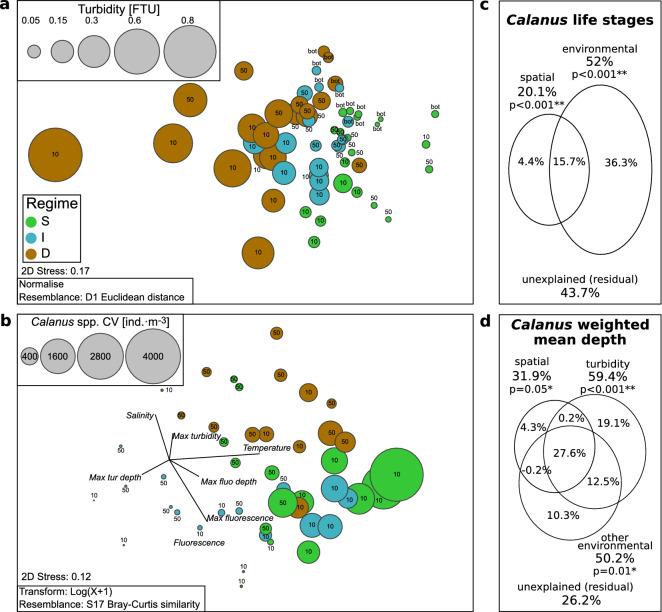
(**a**) nMDS scaling of Euclidian distance resemblance of the environmental variables. The size of the bubbles represents mean turbidity. (**b**) nMDS scaling of Bray–Curtis resemblance of the log-transformed of *Calanus* spp. life stage and nauplii abundances. The size of the bubbles represents the abundance of *Calanus* spp. CV. The color-coding indicates the depth of turbidity change, classified as Shallow (S, green), Intermediate (I, blue) and Deep (D, brown). The vectors indicate the direction of environmental variables at scaled stations (correlation > 0.2 is displayed). Labels indicate sampled water layers (10 for 0–10 m, 50 for 10–50 m and bot for 50–bottom). Venn diagram representing the partition of *Calanus* spp. life stage composition (**c**) and *Calanus* spp. weighted mean depth in the upper 50 m (**d**) explained the variation between the two and three sets of explanatory variables (one or two sets of environmental factors, respectively) and the spatial characteristics of sampling stations (latitude, longitude, and bottom depth).

Despite the fact that ctenophores and gelatinous zooplankton can generally exploit the changing environmental conditions better compared to most other zooplankton groups^81^, their abundance was lower in the inner basin than further offshore^11^. Although gelatinous zooplankton do not directly depend on light availability, the distribution of their prey is the main factor determining their occurrence. The share of *Calanus* spp. CV life stage, which is the most nutritious for predators, was much lower close to the surface in the Deep regime in comparison to the Shallow and Intermediate (Supp. Figs. 3 and 4, Fig. 7). Therefore, dark meltwater plumes could indirectly affect the vertical distribution of non-visual predators by reducing their food base and causing clogging of sticky tentacles by marine snow. Although we did not investigate planktivorous fish or seabirds, other studies showed that most of their diet consisted of *Calanus* spp., especially the CV copepodite stage that is rich in lipids^82–84^. Therefore, the potential influence of *Calanus* spp. distribution and availability at different depth layers can also be of significance for visual predators.

### Comparison of methods

Laser-based optical measurements (LOPC) and imaging (UVP) were well-correlated for studies on *Calanus* spp. (Fig. 5b). Minor differences in comparison with their abundance derived from plankton nets can partially be explained by different seawater volume sampled by these devices. UVP measurements of copepods were also well-correlated with *Calanus* spp. and not with total copepods abundance from the net, most likely due to the fact that UVP does not properly count small mesozooplankton (here dominated by *Oithona similis*) whose size is at the detection limit (500 µm). Also, according to previous studies, the abundance of all particles measured by LOPC in glacial bays was higher than the abundance of zooplankton collected by nets, since LOPC counts fragile aggregates and detritus that are not analyzed in net catches^44,85^. However, in this study, we selected *Calanus*-like particles according to their characteristics, and it seems that they were not affected by the presence of marine snow.

## Conclusions

Here we show that turbid glacial plumes could be one of the important factors responsible for structuring the vertical distribution of *Calanus* spp. during Arctic summer. Moreover, changing light conditions and stratification constraints through the input of turbid glacial meltwater were shown to affect the depth, distribution and intensity of the chlorophyll *a* maximum, which is a proxy for the distribution of preferable food sources for *Calanus* spp. Hence, it seems that these copepods adjust their vertical position to the existing feeding conditions, which in turn is related to turbidity. Here we show that in the medium ranges of turbidity, there might be a slight shift of *Calanus* spp. towards surface waters in comparison to clear open waters at the fjord entrance, whereas in the bays strongly affected by dark glacial plumes, *Calanus* spp. reside deeper within the upper 50 m water layer. We observed diapausing *Calanus* spp. concentrated close to the bottom in all investigated regions and regimes. Our study confirmed that consistent vertical distribution patterns of primarily herbivorous *Calanus* spp. might have been followed by their predators (gelatinous zooplankton). Such significant differences in the distribution of species populations in the Arctic may have important implications for higher (fish, seabirds) or lower (phytoplankton) trophic levels. This study also highlights how optical and/or imaging systems may improve fine-scale observations of distribution patterns of zooplankton in relation to environmental conditions on the compatible scale of measurements. Given the important role of *Calanus* spp. in aquatic food webs, we expect that changes in their communities in relation to turbid plumes will have ramifications in all coastal waters affected by riverine and glacial discharge.

## Supplementary Information

Supplementary Information.

## Data Availability

The datasets generated for this study are available in the Supplementary Material and on request to the corresponding author.
