# Supplementary material for: Dark plumes of glacial meltwater affect vertical distribution of zooplankton in the Arctic
Source: Sci Rep. 2022 Oct 26;12:17953. doi: 10.1038/s41598-022-22475-8 (PMC9606300; doi:10.1038/s41598-022-22475-8)
Supplement: Supplementary file 1 — Supplementary Information. [file 41598_2022_22475_MOESM1_ESM.docx]

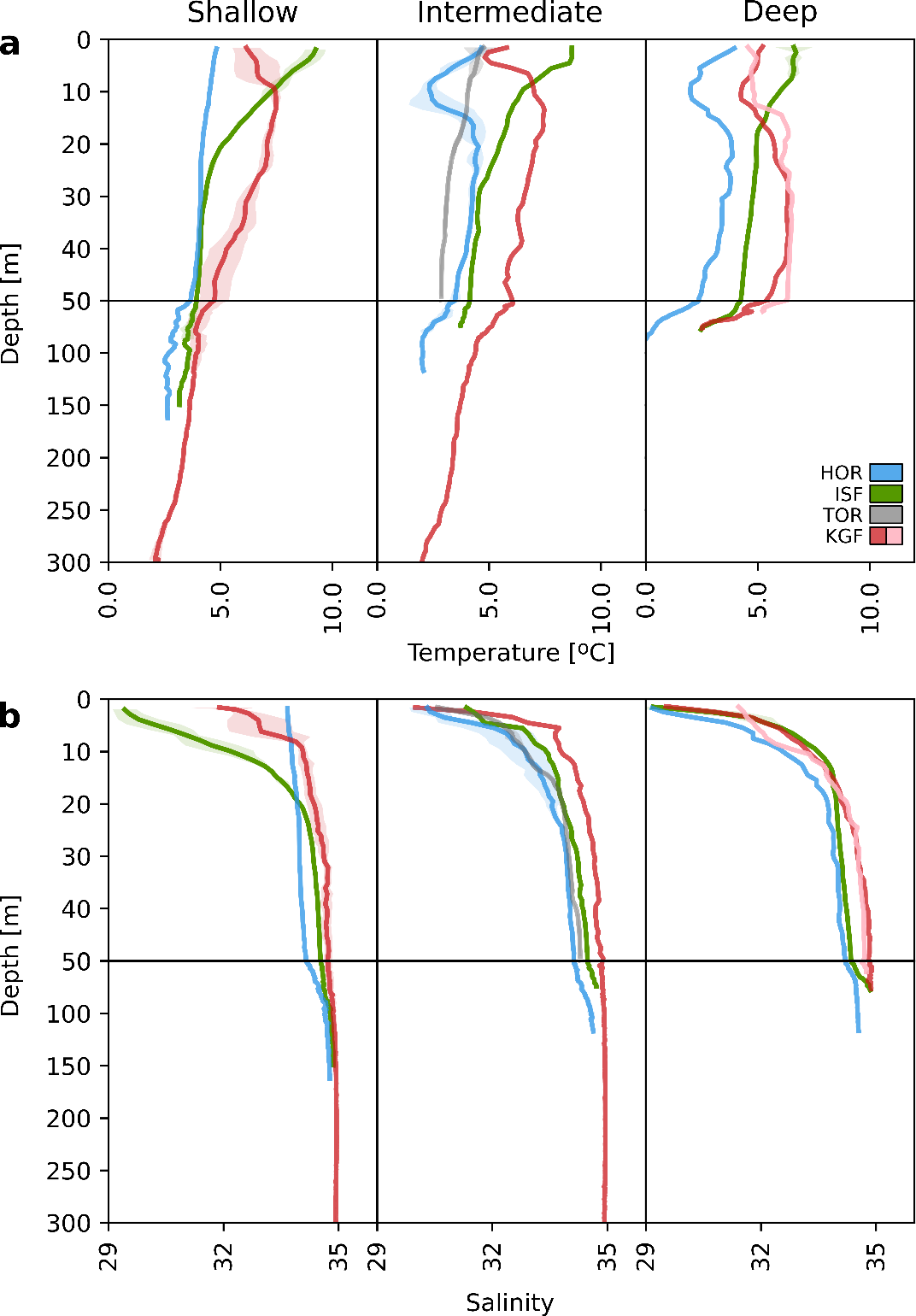


Supplementary Figure 1 Vertical profiles of temperature (°C) and salinity averaged over areas (lines) with standard deviation (shadows) in three regimes of depth of turbidity change. The colour coding indicates regions: Hornsund (HOR, blue), Isfjorden (ISF, green), Torellbreen (TOR, grey), and Kongsfjorden (KGF, red; in the Deep regime: KI and KI2, red; KB5prim and KB5bis, pink). Data for Isfjorden were previously published in Szeligowska et al., 2021.


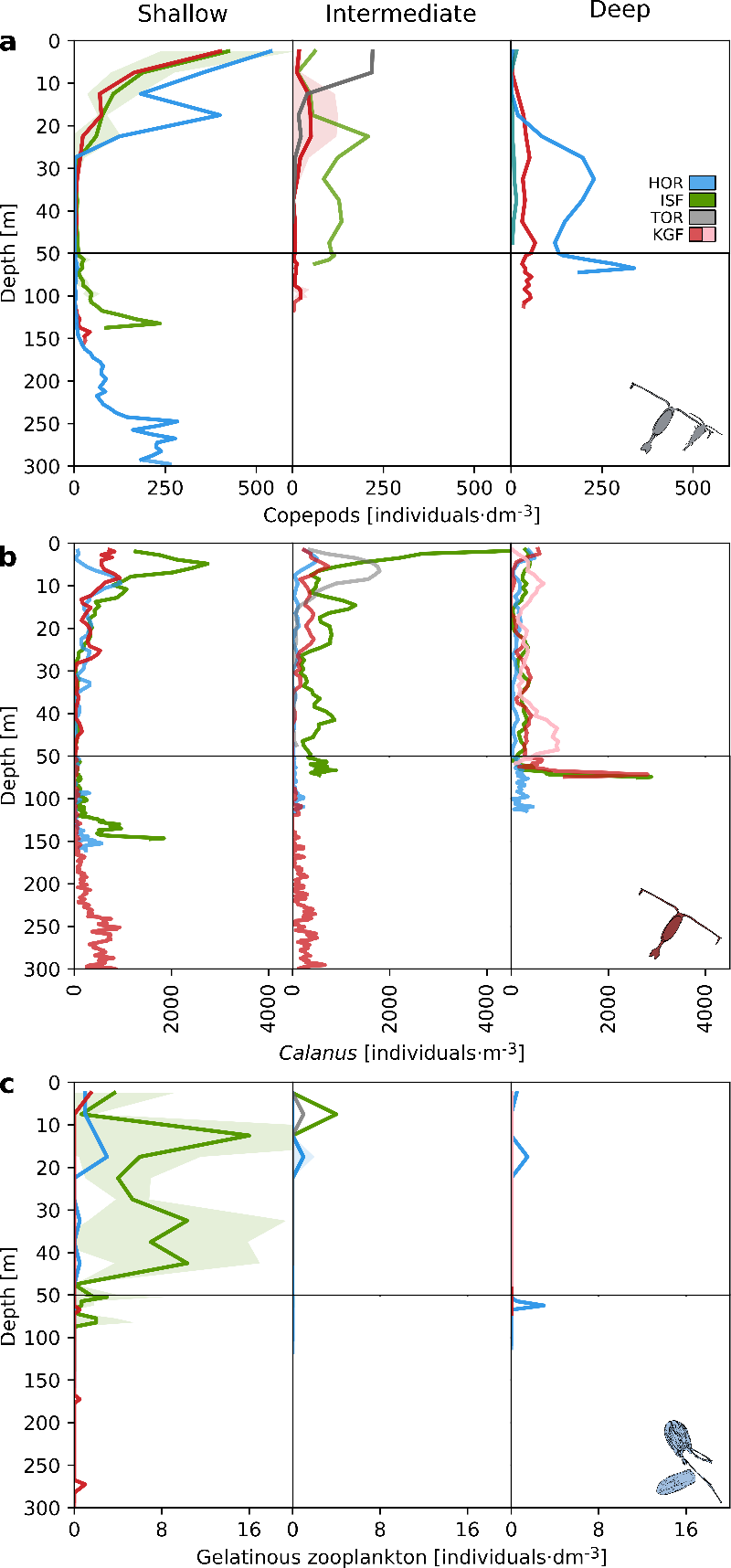


Supplementary Figure 2 Vertical profiles of abundance of copepods (individuals·dm^-3^, UVP), Calanus spp. (individuals·m^-3^, LOPC) and gelatinous zooplankton (individuals·dm^-3^, UVP), averaged over areas (lines) with standard deviation (shadows) in three regimes of depth of turbidity change. The colour coding indicates regions: Hornsund (HOR, red), Isfjorden (ISF, green), Torellbreen (TOR, grey), and Kongsfjorden (KGF, blue; in the Deep regime: KI and KI2, blue; KB5prim and KB5bis, turquoise).


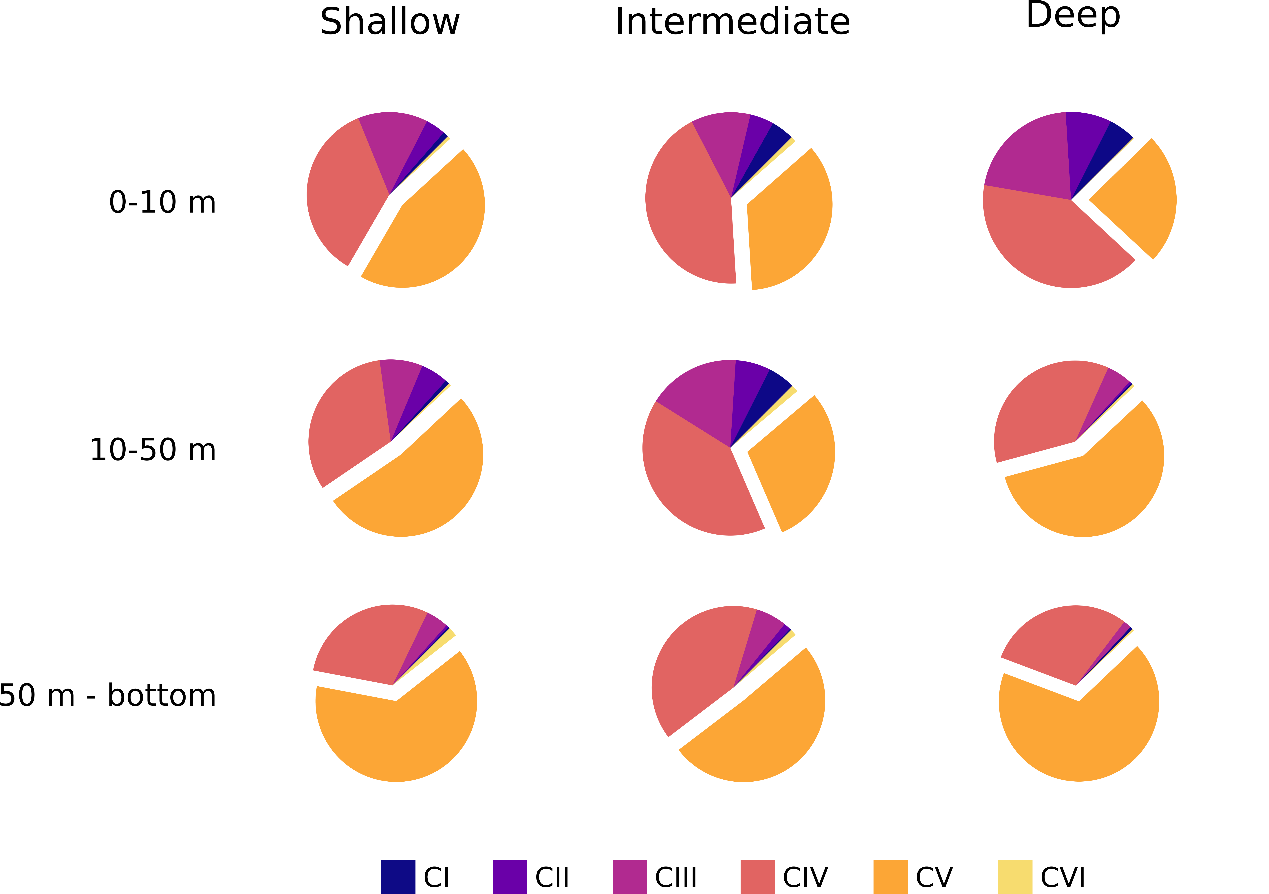


Supplementary Figure 3 Mean shares of life stages of *Calanus* spp. in turbidity regimes and depth layers.


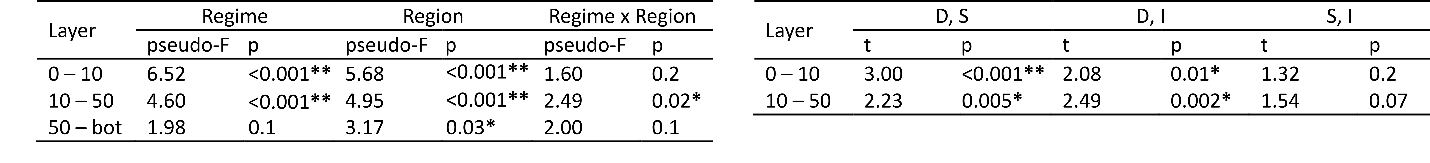


Supplementary Figure 4 Results of PERMANOVA with two factors (regime and region) for three water layers (left), and pair-wise tests for regime (right); ** indicates p<0.001, whereas * indicates p<0.05.

Supplementary Table 1 Environmental parameters: salinity, temperature (°C), turbidity (FTU) and fluorescence of chlorophyll *a* (relative units) averaged for the surface (<10 m), intermediate (10-50 m) and deep (>50 m) water layers and station-specific variables (maximum turbidity, maximum fluorescence and depths for these maxima).

| **Station** | **Salinity** | **Temp.** | **Turbidity** | **Fluoresc.** | **Max turbidity** | **Max fluoresc.** | **Max fluo. depth** | **Max tur. depth** | **Layer** | **Group** | **Region** |
| --- | --- | --- | --- | --- | --- | --- | --- | --- | --- | --- | --- |
| IS1 | 32.08 | 6.45 | 0.21 | 0.10 | 0.39 | 0.18 | 8.50 | 1.28 | 0-10 | D | ISF |
| IS2 | 32.17 | 6.60 | 0.21 | 0.09 | 0.27 | 0.17 | 10.54 | 1.51 | 0-10 | D | ISF |
| IS3 | 32.01 | 6.48 | 0.45 | 0.13 | 1.16 | 0.15 | 10.45 | 1.63 | 0-10 | D | ISF |
| ISF3 | 31.01 | 8.00 | 0.04 | 0.14 | 0.05 | 0.57 | 15.42 | 7.45 | 0-10 | S | ISF |
| IC1 | 30.82 | 8.27 | 0.03 | 0.11 | 0.04 | 0.54 | 17.45 | 2.52 | 0-10 | S | ISF |
| IC2 | 30.11 | 8.75 | 0.04 | 0.13 | 0.05 | 0.58 | 17.47 | 6.55 | 0-10 | S | ISF |
| IA2 | 32.40 | 7.85 | 0.11 | 0.29 | 0.22 | 0.54 | 16.41 | 1.59 | 0-10 | I | ISF |
| KB0 | 33.23 | 7.10 | 0.03 | 0.33 | 0.07 | 0.90 | 21.46 | 1.92 | 0-10 | S | KGF |
| KB1 | 32.68 | 5.62 | 0.10 | 0.19 | 0.11 | 1.07 | 14.39 | 0.98 | 0-10 | S | KGF |
| KI | 32.07 | 4.73 | 0.16 | 0.49 | 0.19 | 0.82 | 6.51 | 11.53 | 0-10 | D | KGF |
| KI2 | 32.01 | 4.85 | 0.30 | 0.68 | 0.42 | 1.10 | 7.56 | 3.46 | 0-10 | D | KGF |
| KB5prim | 32.72 | 4.71 | 0.85 | 0.26 | 1.24 | 0.82 | 3.58 | 7.43 | 0-10 | D | KGF |
| KB5bis | 31.21 | 4.81 | 0.41 | 0.31 | 0.55 | 0.34 | 8.50 | 7.50 | 0-10 | D | KGF |
| H1 | 33.74 | 4.68 | 0.02 | 0.12 | 0.03 | 0.39 | 40.65 | 0.00 | 0-10 | S | HOR |
| HO1 | 33.68 | 4.67 | 0.03 | 0.39 | 0.03 | 0.58 | 18.55 | 3.55 | 0-10 | S | HOR |
| H4 | 31.15 | 3.39 | 0.15 | 0.19 | 0.25 | 0.35 | 9.61 | 12.53 | 0-10 | I | HOR |
| HC1 | 31.66 | 3.48 | 0.16 | 0.28 | 0.24 | 0.87 | 6.57 | 9.49 | 0-10 | I | HOR |
| HC2 | 32.28 | 3.40 | 0.13 | 0.31 | 0.17 | 0.37 | 5.56 | 9.51 | 0-10 | I | HOR |
| H3 | 31.08 | 2.66 | 0.24 | 0.15 | 0.37 | 0.21 | 5.45 | 1.64 | 0-10 | D | HOR |
| H3prim | 31.08 | 2.87 | 0.19 | 0.16 | 0.28 | 0.27 | 5.47 | 4.44 | 0-10 | D | HOR |
| T1 | 32.16 | 4.26 | 0.11 | 0.39 | 0.22 | 0.72 | 12.39 | 1.49 | 0-10 | I | TOR |
| T2 | 32.08 | 4.38 | 0.11 | 0.43 | 0.20 | 0.68 | 14.47 | 1.55 | 0-10 | I | TOR |
| T3 | 32.02 | 4.47 | 0.10 | 0.28 | 0.20 | 0.71 | 13.53 | 1.60 | 0-10 | I | TOR |
| IS1 | 34.09 | 4.76 | 0.14 | 0.06 | 0.39 | 0.18 | 8.50 | 1.28 | 10-50 | D | ISF |
| IS2 | 34.08 | 4.79 | 0.13 | 0.06 | 0.27 | 0.17 | 10.54 | 1.51 | 10-50 | D | ISF |
| IS3 | 34.06 | 4.87 | 0.10 | 0.07 | 1.16 | 0.15 | 10.45 | 1.63 | 10-50 | D | ISF |
| ISF3 | 34.11 | 4.77 | 0.02 | 0.16 | 0.05 | 0.57 | 15.42 | 7.45 | 10-50 | S | ISF |
| IC1 | 34.18 | 4.64 | 0.01 | 0.15 | 0.04 | 0.54 | 17.45 | 2.52 | 10-50 | S | ISF |
| IC2 | 34.10 | 4.76 | 0.02 | 0.15 | 0.05 | 0.58 | 17.47 | 6.55 | 10-50 | S | ISF |
| IA2 | 34.11 | 4.91 | 0.05 | 0.22 | 0.22 | 0.54 | 16.41 | 1.59 | 10-50 | I | ISF |
| KB0 | 34.39 | 6.18 | 0.01 | 0.64 | 0.07 | 0.90 | 21.46 | 1.92 | 10-50 | S | KGF |
| KB1 | 34.46 | 6.29 | 0.01 | 0.49 | 0.11 | 1.07 | 14.39 | 0.98 | 10-50 | S | KGF |
| KI | 34.23 | 5.63 | 0.09 | 0.09 | 0.19 | 0.82 | 6.51 | 11.53 | 10-50 | D | KGF |
| KI2 | 34.60 | 6.04 | 0.06 | 0.12 | 0.42 | 1.10 | 7.56 | 3.46 | 10-50 | D | KGF |
| KB5prim | 34.44 | 6.55 | 0.33 | 0.12 | 1.24 | 0.82 | 3.58 | 7.43 | 10-50 | D | KGF |
| KB5bis | 34.29 | 5.90 | 0.23 | 0.08 | 0.55 | 0.34 | 8.50 | 7.50 | 10-50 | D | KGF |
| H1 | 33.94 | 4.17 | 0.02 | 0.36 | 0.03 | 0.39 | 40.65 | 0.00 | 10-50 | S | HOR |
| HO1 | 34.01 | 4.07 | 0.01 | 0.36 | 0.03 | 0.58 | 18.55 | 3.55 | 10-50 | S | HOR |
| H4 | 33.76 | 3.68 | 0.07 | 0.16 | 0.25 | 0.35 | 9.61 | 12.53 | 10-50 | I | HOR |
| HC1 | 33.70 | 4.06 | 0.04 | 0.28 | 0.24 | 0.87 | 6.57 | 9.49 | 10-50 | I | HOR |
| HC2 | 33.87 | 4.13 | 0.05 | 0.19 | 0.17 | 0.37 | 5.56 | 9.51 | 10-50 | I | HOR |
| H3 | 33.77 | 3.09 | 0.09 | 0.07 | 0.37 | 0.21 | 5.45 | 1.64 | 10-50 | D | HOR |
| H3prim | 33.85 | 3.31 | 0.07 | 0.09 | 0.28 | 0.27 | 5.47 | 4.44 | 10-50 | D | HOR |
| T1 | 33.80 | 3.40 | 0.03 | 0.25 | 0.22 | 0.72 | 12.39 | 1.49 | 10-50 | I | TOR |
| T2 | 33.91 | 3.27 | 0.03 | 0.21 | 0.20 | 0.68 | 14.47 | 1.55 | 10-50 | I | TOR |
| T3 | 33.91 | 3.31 | 0.02 | 0.21 | 0.20 | 0.71 | 13.53 | 1.60 | 10-50 | I | TOR |
| ISF3 | 34.56 | 3.94 | 0.02 | 0.03 | 0.05 | 0.57 | 15.42 | 7.45 | 50-bot | S | ISF |
| IC1 | 34.74 | 3.48 | 0.01 | 0.03 | 0.04 | 0.54 | 17.45 | 2.52 | 50-bot | S | ISF |
| IC2 | 34.74 | 3.47 | 0.02 | 0.03 | 0.05 | 0.58 | 17.47 | 6.55 | 50-bot | S | ISF |
| IA2 | 34.60 | 3.92 | 0.04 | 0.04 | 0.22 | 0.54 | 16.41 | 1.59 | 50-bot | I | ISF |
| KB0 | 34.87 | 3.30 | 0.01 | 0.05 | 0.07 | 0.90 | 21.46 | 1.92 | 50-bot | S | KGF |
| KB1 | 34.90 | 3.36 | 0.01 | 0.04 | 0.11 | 1.07 | 14.39 | 0.98 | 50-bot | S | KGF |
| KI | 34.85 | 4.04 | 0.07 | 0.03 | 0.19 | 0.82 | 6.51 | 11.53 | 50-bot | D | KGF |
| H1 | 34.57 | 2.86 | 0.02 | 0.06 | 0.03 | 0.39 | 40.65 | 0.00 | 50-bot | S | HOR |
| HO1 | 34.64 | 2.80 | 0.01 | 0.05 | 0.03 | 0.58 | 18.55 | 3.55 | 50-bot | S | HOR |
| H4 | 34.42 | 2.33 | 0.02 | 0.04 | 0.25 | 0.35 | 9.61 | 12.53 | 50-bot | I | HOR |
| HC1 | 34.39 | 2.46 | 0.02 | 0.05 | 0.24 | 0.87 | 6.57 | 9.49 | 50-bot | I | HOR |
| HC2 | 34.31 | 2.65 | 0.02 | 0.05 | 0.17 | 0.37 | 5.56 | 9.51 | 50-bot | I | HOR |
| H3 | 34.45 | 0.45 | 0.04 | 0.02 | 0.37 | 0.21 | 5.45 | 1.64 | 50-bot | D | HOR |
| H3prim | 34.46 | 0.40 | 0.03 | 0.02 | 0.28 | 0.27 | 5.47 | 4.44 | 50-bot | D | HOR |

Supplementary Table 2 *Calanus* spp. life stage and nauplii abundances (individuals·m^-3^) in the three water layers.

| **Station** | **VI** | **V** | **IV** | **III** | **II** | **I** | **nauplii** | **Layer** | **Regime** | **Region** |
| --- | --- | --- | --- | --- | --- | --- | --- | --- | --- | --- |
| IS1 | 0.00 | 304.00 | 136.00 | 40.00 | 0.00 | 2.64 | 8.00 | 0-10 | D | ISF |
| IS2 | 0.00 | 208.00 | 232.00 | 48.00 | 8.00 | 8.00 | 0.00 | 0-10 | D | ISF |
| IS3 | 0.00 | 432.00 | 368.00 | 5.28 | 5.28 | 0.00 | 0.00 | 0-10 | D | ISF |
| ISF3 | 13.20 | 2520.00 | 1840.00 | 480.00 | 40.00 | 0.00 | 0.00 | 0-10 | S | ISF |
| IC1 | 0.00 | 912.00 | 544.00 | 176.00 | 5.28 | 5.28 | 0.00 | 0-10 | S | ISF |
| IC2 | 0.00 | 1260.00 | 1060.00 | 380.00 | 80.00 | 6.60 | 0.00 | 0-10 | S | ISF |
| IA2 | 0.00 | 512.00 | 960.00 | 256.00 | 48.00 | 16.00 | 0.00 | 0-10 | I | ISF |
| KB0 | 93.32 | 3760.00 | 1520.00 | 440.00 | 13.32 | 0.00 | 0.00 | 0-10 | S | KGF |
| KB1 | 37.33 | 672.00 | 272.00 | 160.00 | 128.00 | 48.00 | 64.00 | 0-10 | S | KGF |
| KI | 0.00 | 2.80 | 12.80 | 13.60 | 4.40 | 2.40 | 336.00 | 0-10 | D | KGF |
| KI2 | 1.33 | 8.00 | 88.00 | 132.00 | 72.00 | 52.00 | 480.00 | 0-10 | D | KGF |
| KB5prim | 3.33 | 580.00 | 1033.33 | 190.00 | 10.00 | 3.33 | 50.00 | 0-10 | D | KGF |
| KB5bis | 3.20 | 6.40 | 70.40 | 102.40 | 57.60 | 41.60 | 32.00 | 0-10 | D | KGF |
| H1 | 0.00 | 48.00 | 272.00 | 144.00 | 72.00 | 16.00 | 56.00 | 0-10 | S | HOR |
| HO1 | 0.00 | 720.00 | 789.28 | 288.00 | 5.28 | 5.28 | 48.00 | 0-10 | S | HOR |
| H4 | 6.00 | 28.00 | 48.00 | 10.00 | 8.00 | 14.00 | 116.00 | 0-10 | I | HOR |
| HC1 | 2.64 | 200.00 | 160.00 | 88.00 | 24.00 | 40.00 | 184.00 | 0-10 | I | HOR |
| HC2 | 2.00 | 60.00 | 72.00 | 22.00 | 8.00 | 14.00 | 116.00 | 0-10 | I | HOR |
| H3 | 0.00 | 1.20 | 14.40 | 7.20 | 2.80 | 1.60 | 24.00 | 0-10 | D | HOR |
| H3prim | 0.00 | 8.00 | 35.20 | 16.80 | 6.40 | 0.80 | 0.00 | 0-10 | D | HOR |
| T1 | 0.00 | 944.00 | 672.00 | 160.00 | 80.00 | 0.00 | 5.28 | 0-10 | I | TOR |
| T2 | 0.00 | 768.00 | 864.00 | 133.28 | 80.00 | 32.00 | 16.00 | 0-10 | I | TOR |
| T3 | 6.60 | 780.00 | 1360.00 | 260.00 | 100.00 | 0.00 | 40.00 | 0-10 | I | TOR |
| IS1 | 0.00 | 212.00 | 160.00 | 8.00 | 0.00 | 4.00 | 0.00 | 10-50 | D | ISF |
| IS2 | 0.00 | 200.00 | 92.50 | 2.50 | 0.00 | 0.00 | 0.00 | 10-50 | D | ISF |
| IS3 | 0.00 | 118.36 | 51.68 | 3.33 | 0.00 | 0.55 | 5.00 | 10-50 | D | ISF |
| ISF3 | 1.30 | 76.00 | 52.00 | 12.00 | 4.00 | 0.00 | 0.00 | 10-50 | S | ISF |
| IC1 | 0.00 | 64.00 | 46.00 | 16.00 | 4.00 | 0.00 | 0.00 | 10-50 | S | ISF |
| IC2 | 0.00 | 104.00 | 66.00 | 12.00 | 18.00 | 0.00 | 8.00 | 10-50 | S | ISF |
| IA2 | 1.98 | 246.00 | 306.00 | 84.00 | 12.00 | 18.00 | 6.00 | 10-50 | I | ISF |
| KB0 | 3.33 | 1120.00 | 120.00 | 60.00 | 40.00 | 3.33 | 20.00 | 10-50 | S | KGF |
| KB1 | 8.00 | 180.00 | 100.00 | 53.28 | 72.00 | 20.00 | 48.00 | 10-50 | S | KGF |
| KI | 1.67 | 120.00 | 320.00 | 125.00 | 0.00 | 0.00 | 0.00 | 10-50 | D | KGF |
| KI2 | 3.33 | 800.00 | 320.00 | 60.00 | 3.33 | 0.00 | 0.00 | 10-50 | D | KGF |
| KB5prim | 23.33 | 550.00 | 430.00 | 50.00 | 10.00 | 0.00 | 0.00 | 10-50 | D | KGF |
| KB5bis | 8.89 | 297.85 | 133.33 | 13.33 | 0.00 | 1.48 | 13.33 | 10-50 | D | KGF |
| H1 | 1.32 | 124.00 | 100.00 | 20.00 | 8.00 | 0.00 | 20.00 | 10-50 | S | HOR |
| HO1 | 1.65 | 275.00 | 350.00 | 55.00 | 1.65 | 5.00 | 15.00 | 10-50 | S | HOR |
| H4 | 1.00 | 6.50 | 11.50 | 6.00 | 4.00 | 5.50 | 7.50 | 10-50 | I | HOR |
| HC1 | 1.32 | 60.00 | 188.00 | 29.32 | 12.00 | 16.00 | 68.00 | 10-50 | I | HOR |
| HC2 | 0.00 | 9.00 | 10.00 | 11.50 | 2.00 | 1.00 | 8.00 | 10-50 | I | HOR |
| H3 | 0.00 | 135.00 | 62.50 | 5.00 | 5.00 | 0.00 | 12.50 | 10-50 | D | HOR |
| H3prim | 0.00 | 72.00 | 46.00 | 4.00 | 0.00 | 2.00 | 14.00 | 10-50 | D | HOR |
| T1 | 1.00 | 12.00 | 30.00 | 16.00 | 8.00 | 1.00 | 100.00 | 10-50 | I | TOR |
| T2 | 1.00 | 28.00 | 36.00 | 8.00 | 2.00 | 0.00 | 92.00 | 10-50 | I | TOR |
| T3 | 2.05 | 35.90 | 14.36 | 8.21 | 5.13 | 5.13 | 71.79 | 10-50 | I | TOR |
| ISF3 | 0.00 | 35.00 | 39.00 | 6.00 | 0.33 | 0.00 | 4.00 | 50-bot | S | ISF |
| IC1 | 0.73 | 124.44 | 57.78 | 2.22 | 2.22 | 0.00 | 2.22 | 50-bot | S | ISF |
| IC2 | 0.66 | 160.00 | 60.66 | 4.00 | 2.00 | 0.00 | 2.00 | 50-bot | S | ISF |
| IA2 | 16.00 | 352.00 | 280.00 | 32.00 | 8.00 | 8.00 | 32.00 | 50-bot | I | ISF |
| KB0 | 3.52 | 298.30 | 86.40 | 2.13 | 0.00 | 0.00 | 9.60 | 50-bot | S | KGF |
| KB1 | 1.59 | 289.05 | 97.75 | 27.93 | 4.65 | 4.65 | 23.27 | 50-bot | S | KGF |
| KI | 10.66 | 856.16 | 224.00 | 16.00 | 0.00 | 0.00 | 0.00 | 50-bot | D | KGF |
| H1 | 6.12 | 53.18 | 11.30 | 0.87 | 0.00 | 0.00 | 6.96 | 50-bot | S | HOR |
| HO1 | 0.34 | 6.06 | 4.88 | 1.68 | 0.00 | 0.17 | 0.84 | 50-bot | S | HOR |
| H4 | 0.37 | 21.28 | 9.20 | 0.13 | 0.00 | 0.00 | 3.20 | 50-bot | I | HOR |
| HC1 | 0.12 | 20.37 | 19.20 | 2.95 | 0.12 | 0.00 | 2.46 | 50-bot | I | HOR |
| HC2 | 0.36 | 9.24 | 11.47 | 3.20 | 1.07 | 0.00 | 7.11 | 50-bot | I | HOR |
| H3 | 0.34 | 110.98 | 51.29 | 0.34 | 0.00 | 0.00 | 1.03 | 50-bot | D | HOR |
| H3prim | 0.00 | 1152.00 | 736.16 | 64.00 | 0.00 | 32.00 | 224.00 | 50-bot | D | HOR |
